# Pioglitazone ameliorates the phenotype of a novel Parkinson’s disease mouse model by reducing neuroinflammation

**DOI:** 10.1186/s13024-016-0090-7

**Published:** 2016-04-02

**Authors:** Milena Pinto, Nadee Nissanka, Susana Peralta, Roberta Brambilla, Francisca Diaz, Carlos T. Moraes

**Affiliations:** Department of Neurology, University of Miami Miller School of Medicine, 1420 NW 9th Avenue, Rm.229, Miami, FL 33136 USA; Neuroscience Graduate Program, University of Miami Miller School of Medicine, Miami, FL 33136 USA; The Miami Project To Cure Paralysis, University of Miami Miller School of Medicine, Miami, FL 33136 USA; Department of Cell Biology, University of Miami Miller School of Medicine, Miami, FL 33136 USA

**Keywords:** Mitochondria, Pioglitazone, Neuroinflammation

## Abstract

**Background:**

Parkinson’s disease (PD) is a progressive neurodegenerative disorder characterized by motor and non-motor symptoms. The cause of the motor symptoms is the loss of dopaminergic neurons in the *substantia nigra* with consequent depletion of dopamine in the striatum. Although the etiology of PD is unknown, mitochondrial dysfunctions, including cytochrome c oxidase (Complex IV) impairment in dopaminergic neurons, have been associated with the disease’s pathophysiology. In order to analyze the role of Complex IV in PD, we knocked out *Cox10* (essential for the maturation of COXI, a catalytic subunit of Complex IV) in dopaminergic neurons. We also tested whether the resulting phenotype was improved by stimulating the PPAR-γ pathway.

**Results:**

*Cox10*/DAT-cre mice showed decreased numbers of TH+ and DAT+ cells in the *substantia nigra*, early striatal dopamine depletion, motor defects reversible with L-DOPA treatment and hypersensitivity to L-DOPA with hyperkinetic behavior. We found that chronic pioglitazone (PPAR-γ agonist) treatment ameliorated the motor phenotype in *Cox10*/DAT-cre mice. Although neither mitochondrial function nor the number of dopaminergic neurons was improved, neuroinflammation in the midbrain and the striatum was decreased.

**Conclusions:**

By triggering a mitochondrial Complex IV defect in dopaminergic neurons, we created a new mouse model resembling the late stages of PD with massive degeneration of dopaminergic neurons and striatal dopamine depletion. The motor phenotypes were improved by Pioglitazone treatment, suggesting that targetable secondary pathways can influence the development of certain forms of PD.

**Electronic supplementary material:**

The online version of this article (doi:10.1186/s13024-016-0090-7) contains supplementary material, which is available to authorized users.

## Background

Parkinson’s disease (PD) is characterized by non-motor and motor symptoms, the latter caused by a depletion of striatal dopamine (DA) due to neurodegeneration of dopaminergic neurons in the *substantia nigra* (SN). Numerous studies show that mitochondrial dysfunction plays an important role in the pathophysiology of PD and that complexes of the mitochondrial electron transport chain are affected [1, 2].

Different laboratories have analyzed the activity of the mitochondrial respiratory chain complexes in samples of PD, but the results were variable. Complex I activity was decreased in the SN, platelets and muscles of affected individuals [3] and SN dopaminergic neurons from PD patients harbored high levels of mtDNA deletions and cytochrome c oxidase dysfunction [4, 5]. Some groups found impaired complex II, complex II + III, and complex IV activity in platelets [6], whereas others reported a selective complex I inhibition [3]. Different studies also showed impaired complex I, II + III, and IV in PD muscle [7–9], whereas others reported no differences, and results on PD lymphocytes are even more contradictory.

A small percentage of PD cases is monogenic and investigations into the associated gene mutations confirmed the importance of mitochondria. PARK2 (encoding for Parkin) and PINK1, for example, are two genes mutated in rare forms of monogenic PD, and they cooperate for selective autophagy of damaged mitochondria (mitophagy) [10, 11].

Because mitochondrial dysfunctions appear to have a role in the pathogenesis of PD, it has been suggested that an increase in neuronal mitochondrial content could compensate for the bioenergetic defects that lead to neurodegeneration [12, 13].

Pioglitazone is an agonist of PPAR-γ (peroxisome proliferator-activated receptor γ), a receptor that regulates cellular functions such as lipid metabolism, cell growth, differentiation and inflammation. PPAR-γ is co-activated by PGC-1α a master regulator of mitochondrial biogenesis [14]. Pioglitazone treatment has been shown to increase mitochondrial biogenesis in various tissues and to reduce neurodegeneration in different mouse models. In an X-linked adrenoleukodystrophy model, pioglitazone restored mitochondrial content and locomotor impairment [15]. In Alzheimer’s disease models, it improved spatial learning, enhanced AKT signaling, and attenuated tau hyperphosphorylation and neuroinflammation [16], probably by enhancing the microglial uptake of beta-amyloid [17, 18]. Pioglitazone ameliorated the 3-nitropropionic acid-induced mitochondrial dysfunction in striatal neurons in a mouse model of Huntington’s disease [19]. It has also been used on acute pharmacological models of PD, attenuating neurodegeneration in MPTP-treated mice, and monkeys [20–23]. Here we describe that pioglitazone can ameliorate the motor symptoms of a novel genetic mouse model of PD not by preventing dopaminergic neuron loss but by reducing inflammation.

## Results

### Generation of a novel PD mouse model: *Cox10*/DAT-cre mice

*Cox10* encodes for a heme-*o*-farnesyl transferase involved in heme-*a* biosynthesis, which is essential for the maturation of CoxI, one of the catalytic subunit of cytochrome *c* oxidase (Complex IV of the mitochondrial electron transport chain) [24]. In order to knock out *Cox10* only in dopaminergic neurons, homozygous *Cox10* floxed mice (*Cox10*^f/f^) [25] were bred with transgenic mice expressing cre recombinase under the control of the dopamine transporter (*DAT-Scl6a3*) promoter [26]. *Cox10*^f/f^-*DAT-cre*^+^ mice (*Cox10*/DAT-cre) were used as experimental animals and were compared to DAT-cre control animals (*Cox10*^wt/wt^-*DAT-cre*^+^).

**Fig. 1:**
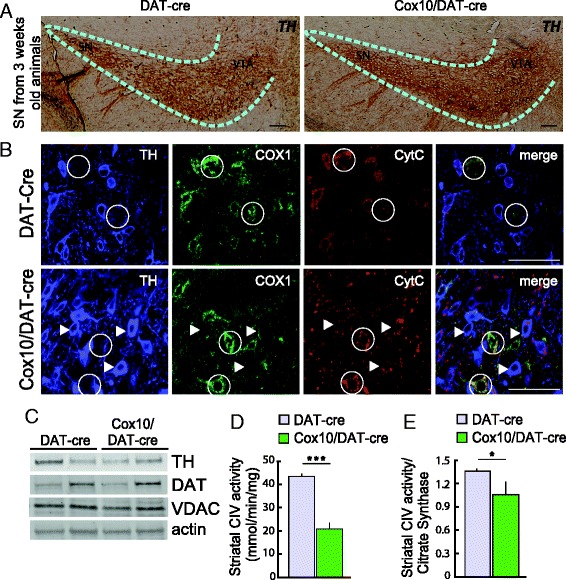
Cox deficiency in dopaminergic circuit of *Cox10*/DAT-cre mice. **a** Immunohistochemical images of TH staining on midbrain sections of three-week-old animals show no apparent difference in cell number between DAT-cre and *Cox10*/DAT-cre mice. (SN: *substantia nigra*, VTA: ventral tegmental area). **b** triple staining (TH-COX1-CytC) on midbrain sections of one-month-old control and Cox10 deficient mice. In control mice all the TH+ cells are also COX1+ and CytC+. Some TH- COX+ CytC+ cells are present in the region. These cells are still present in *Cox10*/DAT-cre mice sections (in white circles), while TH+ neurons are negative for COX1 (white arrows), indicating a selective deletion of *Cox10* gene in dopaminergic cells. **c** Western blots in striatal homogenates of DAT-cre and *Cox10*/DAT-cre mice show no changes in TH, DAT and VDAC-porin levels (actin for normalization). **d**-**e** Complex IV enzymatic activity on striatal samples of one-month-old DAT-cre (lilac) and *Cox10*/DAT-cre (green) mice normalized to mg of protein **d** or to Citrate synthase **e** (*n* = 3/group)

The two groups of mice were born at Mendelian ratios, with a normal lifespan (up to 24 months of age). Because *Cox10* deletion starts during embryonic development, we analyzed TH+ cells in the *substantia nigra* of three-week-old animals and found no reductions in TH+ cells in *Cox10*/DAT-cre animals at this young age (Fig. 1a).

Because there are no good antibodies available for Cox10, we detected CoxI as a surrogate: in the absence of Cox10, COXI is unstable because it cannot incorporate heme-*a*, leading to its degradation. To verify that *Cox10* deletion occurred specifically in dopaminergic neurons, we performed triple immunohistochemistry on the midbrain of one-month-old animals with antibodies against COXI, TH (for dopaminergic neurons) and cytochrome *c* (mitochondria) (Fig. 1b). In *Cox10*/DAT-cre mice most of the dopaminergic neurons (TH+) lacked CoxI staining (Fig. 1b, white arrows) while TH negative cells present in the midbrain were still positive for COXI (Fig. 1b, white circles), demonstrating the specificity of the *Cox10* deletion.

Because the dopaminergic neurons that form the nigrostriatal pathway project their axons to the striatum, we analyzed TH and DAT protein expression in the striatum of three-week-old animals. We found no differences between *Cox10*/DAT-cre and DAT-cre mice, indicating absence of axonal degeneration at this age (Fig. 1c). We also found no difference in VDAC (marker of mitochondrial mass) levels.

To analyze Complex IV (CIV) activity in this region, we performed an enzymatic assay on homogenates from striata of one-month-old animals. *Cox10*/DAT-cre mouse showed, as expected, a decrease in CIV activity of approximately 50 % compared to control mice (Fig. 1d). When CIV activity was normalized to Citrate synthase (CS) (enzyme localized in the mitochondrial matrix) the reduction was of 22 % (Fig. 1e).

### *Cox10*/DAT-cre mice showed progressive motor impairment, which was reverted by L-DOPA treatment

**Fig. 2:**
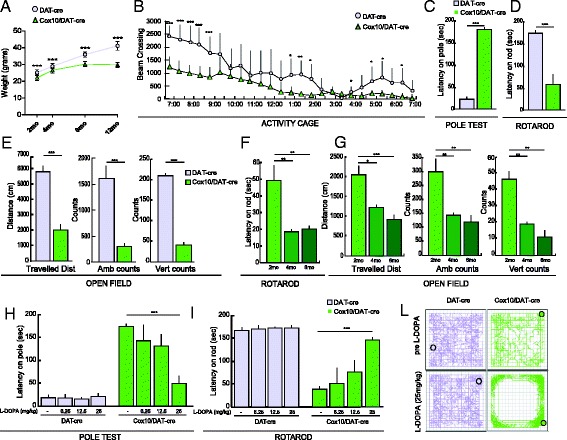
*Cox10* deficient mice display abnormal motor behaviors at 2 months of age. **a** Body weight measurement of 2, 4, 8 and 12-month-old animals: *Cox10*/DAT-cre mice (green triangles) showed a significant reduction in weight compared to age matched DAT-cre mice (lilac circles) (*n* = 10-15). **b** Activity cage: two-month-old *Cox10*/DAT-cre mice have less exploratory behavior and spontaneous activity compared to DAT-cre animals (*n* = 8). **c**-**d** Pole test **c** and rotarod **d** performed by 2-month-old *Cox10*/DAT-cre and DAT-cre mice. *Cox10*/DAT-cre mice show less coordination, not been able to descend the pole and falling from the road after less than one minute (*n* = 10). **e** Open field test: *Cox10*/DAT-cre mice show a reduced voluntary movement with decreased travelled distance and a decreased ambulatory and rearing activity compared to DAT-cre animals (*n* = 7); **f**-**g** Worsening of the motor phenotype in 4-month-old animals. 4 and 6-month-old *Cox10*/DAT-cre mice perform worse on the rotarod **f** and show reduced voluntary movement compared to 2-month-old animals **g**. **h**-**i** Pole test **h** and rotarod **i** performed by 2-month-old *Cox10*/DAT-cre mice before and after increasing dosage of L-DOPA (*n* = 4). Motor defects were rescued by the higher L-DOPA dosage. **l**: representative image of route travelled by a mouse before (upper panel) and one hour after injection with the highest dosage (25 mg/kg) of L-DOPA (lower panel)

*Cox10*/DAT-cre mice were born with a slightly but significantly lower body weight when compared to controls. This difference was maintained throughout life (Fig. 2a). The major symptom resulting from the disruption of the nigrostriatal pathway in PD patients is the decline in motor coordination. In order to detect any motor phenotype, we tested the mice in open field and activity cage to analyze general motor activity, and in pole test and Rotarod to assess motor coordination.

At 2 months of age, *Cox10*/DAT-cre mice showed a significant decline in motor activity and coordination, moving less during the nocturnal cycle (Fig. 2b) and in the open field (Fig 2e). *Cox10*/DAT-cre mice showed decreased Travelled Distance, Ambulatory Counts as well as rearing activity (Vertical Counts) (Fig. 2e). When tested for motor coordination, *Cox10*/DAT-cre mice were not able to perform the pole test (Fig. 2c) and their performance on the Rotarod was impaired compared to age-matched controls (Fig. 2d).

When tested at different ages, we noticed that mice reached the maximal decline in motor coordination by the age of 4 months, with no further worsening by 6 months (Fig. 2f-g). Compared to younger animals, 4 and 6-months old *Cox10*/DAT-cre mice showed a decreased walking time on the rotarod (about 18 and 20 seconds, compared to 49 seconds of younger animals) (Fig. 2f) as well as decreased travelled distance, ambulatory counts and rearing activity in the open field, (Fig. 2g). Most of the mice were not able to descend the pole within a given maximum time of 180 s (data not shown).

To ensure that the motor impairment was due to a defect in the dopaminergic system, we performed the same behavioral analyses after administration of L-3,4-dihydroxyphenylalanine (L-DOPA), the drug used in PD patients to treat motor symptoms. Three different groups of 2-month old mice were injected I.P. with different doses (6.25, 12.5 and 25 mg/kg) of L-DOPA in combination with the peripheral DOPA decarboxylase inhibitor benserazide and tested for motor coordination pre-injection and 1 hour post-injection.

The low L-DOPA doses (6.25 and 12.5 mg/kg) were not sufficient to rescue the motor phenotype of *Cox10*/DAT-cre, whereas the higher dose (25 mg/kg) improved the behavioral outcome in both the pole test and the Rotarod (Fig. 2h, i), indicating that the motor phenotype is due to the depletion of dopamine in the striatum. Noticeably, after administration of the 25 mg/kg dose, we also measured hyperactivity in the *Cox10*/DAT-cre mice, with an intense bout of locomotion and stereotypic movements (Fig. 2l).

### Striatal alterations and neurodegeneration in the *Cox10*/DAT-cre mice

**Fig. 3:**
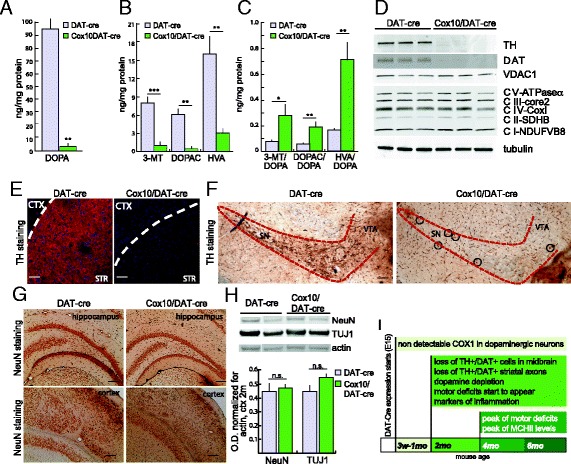
Early loss of tyrosine hydroxylase positive neurons in the *Cox10*/DAT-cre mice. **a** dopamine quantification from striatal homogenates of 2-month-old animals normalized to mg of protein. **b** quantification of 3-MT, DOPAC and HVA from striatal homogenates normalized to mg of protein. **c** ratio of dopamine metabolites to dopamine content. (*n* = 4/group). This analysis shows drastic dopamine depletion in striata of *Cox10*/DAT-cre mice, as well as a decrease in dopamine metabolites. **d** Western blotting probing for TH and DAT in representative striatal homogenates of DAT-cre and *Cox10*/DAT-cre mice shows an undetectable presence of dopaminergic markers (actin for normalization). No changes in mitochondrial markers were detected (VDAC-porin, NDUFB8 (CI subunit), SDHB (CII subunit), UQCRC2 (core2 subunit of CIII), COXI (CIV subunit) and ATPase-α (CV subunit) (*n* = 4/group). **e** Representative immunohistochemical staining of TH in striata of 2-month-old animals (CTX = cortex, STR = striatum). **f** Representative immunohistochemical identification of TH+ neurons in midbrain sections of DAT-cre and *Cox10*/DAT-cre mice (black circles depict rare TH+ neuronal bodies). A massive neurodegeneration is already present at 2 months of age in *Cox10*/DAT-cre mice. **g** Immunohistochemical staining with anti-NeuN of hippocampus and cortex sections of 2-month-old DAT-cre and *Cox10*/DAT-cre mice shows no neurodegeneration in these regions. **h**: Western blot and relative quantification probing for NeuN and TUJ1 in cortical homogenates of 2-month-old DAT-cre and *Cox10*/DAT-cre mice. No changes in neuronal markers were detected in these regions. **i**: graphical representation of pathology progression of *Cox10*/DAT-cre mice. Scale bar = 50 μm. In all figures, error bars represent SEM. *p* values are indicated by asterisks (*p < 0.05, **p < 0.01, ***p < 0.001)

In PD patients, the direct cause of motor symptoms is the depletion of striatal DA caused by neurodegeneration of dopaminergic neurons of the SN. Because the motor phenotypes in *Cox10*/DAT-cre mice were evident at early time points, we analyzed the effects of the absence of *Cox10* in 2-month-old animals. We analyzed the levels of DA and DA metabolites in the striatum of *Cox10*/DAT-cre mice and found a marked decrease in DA content compared to controls (Fig. 3a). DA metabolites, (dihydroxyphenylacetic acid (DOPAC), 3-methoxytyramine (3-MT) and homovanillic acid (HVA)) were also decreased (Fig. 3b) but to a lesser extent. As a result, the ratio between DA metabolites and DA was increased in *Cox10*/DAT-cre mice compared to controls (Fig. 3c), which is usually a sign of impaired regulation of DA metabolism. The massive depletion in DA and its metabolites content was maintained but not exacerbated during the lifetime, at 4, 8 and 12 months (data not shown).

To analyze if the severe depletion of DA was due to axonal degeneration, we measured the content of the dopaminergic-specific proteins tyrosine hydroxylase (TH) and dopamine transporter (DAT) in the striatum. We found undetectable levels of both dopaminergic markers in *Cox10*/DAT-cre mice compared to controls (Fig. 3d), suggesting widespread axonal degeneration, which was further confirmed by IHC for TH (Fig. 3e) and DAT (data not shown). We also analyzed the steady-state level of several mitochondrial proteins, including VDAC-porin, NDUFB8 (CI subunit), SDHB (CII subunit), UQCRC2 (CIII subunit), COXI (CIV subunit) and ATPase-α (CV subunit) in striatal homogenates (Fig. 3d), but found no changes, indicating that the residual striatal cell content, which by then had lost most dopaminergic axons, had no mitochondrial defects.

In previously described mouse models [27, 28], the degeneration of dopaminergic neurons was suggested to start from the axons and proceed towards the cell body. To analyze if dopaminergic cell bodies were still present in our model at 2 months of age, we examined frontal sections, including the midbrain region, of *Cox10*/DAT-cre animals for TH-positive dopaminergic neurons. Two-month-old animals already showed a massive reduction of TH content, suggesting a degeneration in the SN, with only few TH positive cells remaining (Fig. 3f). Overlapping results were obtained using anti-DAT antibody as a second marker of dopaminergic cells (data not shown).

To demonstrate that the neurodegeneration was specific for dopaminergic neurons, we performed IHC (Fig. 3g) and western blot (Fig. 3h) in different brain regions probing for two different neuronal markers (NeuN and TUJ1). As expected, we found no significant differences between *Cox10*/DAT-cre animals and age-matched controls. Figure 3i illustrates the phenotypic changes over time.

### Pioglitazone treatment improved motor function of the *Cox10*/DAT-cre mice

Increase in mitochondrial biogenesis has been reported to be beneficial in models of oxidative phosphorylation (OXPHOS) deficiencies [29, 30]. In order to analyze the effect of chronic pioglitazone treatment on motor behavior in our mouse model, we administered the drug by incorporating it into the food (120 mg/kg). We measured food intake and found no difference between DAT-Cre (3.61 ± 0.06 g/day) and *Cox10*/DAT-Cre (3.80 ± 0.07 g/day) animals, even though both ate less than regular C57BL/6 J wt animals (4.75 ± 0.07 g/day). We estimated the dosage to be approximately 16 mg/kg per day. Even if only 18 % of pioglitazone crosses the blood–brain-barrier, as previously estimated [31], the dose is in the pharmacologically active range.

**Fig. 4:**
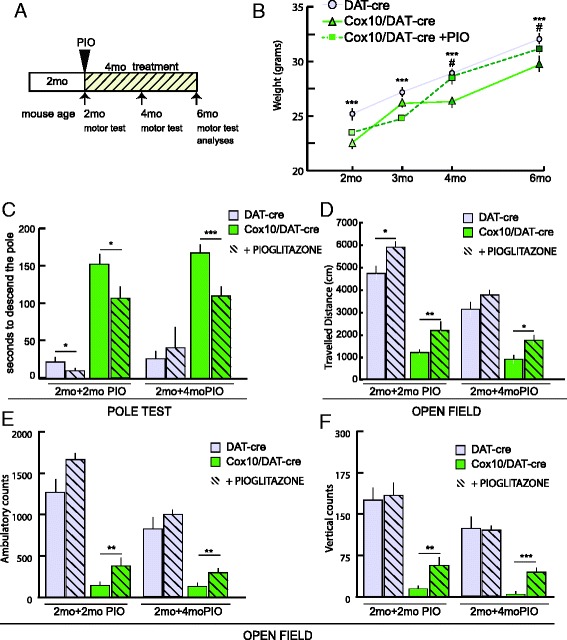
Pioglitazone partially rescues motor behavior in *Cox10*/DAT-cre mice after 4 months of treatment. **a** Schematic representation of pioglitazone treatment and experimental time points. **b** Body weight measurement of 2, 3, 4 and 6-month-old animals: after two months of pioglitazone treatment (4-month-old animals) (dashed line) *Cox10*/DAT-cre mice showed a significant increase in weight compared to *Cox10*/DAT-cre mice fed with standard diet (green continuous line) (*n* = 4). ***p < 0.001: DAT-cre compared to *Cox10*/DAT-cre; #*p* < 0.05: *Cox10*/DAT-cre untreated and treated with Pioglitazone. **c** Pole test: *Cox10*/DAT-cre mice displayed decreased latencies times when required to descend a pole, after pioglitazone treatment (*n* = 4). **d**, **e**, **f** Open field test: *Cox10*/DAT-cre mice show an increased voluntary movement **d**, **e** and an increased rearing activity **f** after pioglitazone treatment (*n* = 4)

Two-month old *Cox10*/DAT-cre and control mice were fed with pioglitazone-chow for 4 months. Motor behavior was analyzed at the beginning of the treatment, then after 2 and 4 months (4 and 6 months of age) (Fig. 4a). We monitored body weight throughout the treatment. *Cox10*/DAT-cre mice weighted less than controls at all ages (Figs. 2a and 4b). After 2 months of treatment, the weight of *Cox10*/DAT-cre mice was comparable to untreated controls (Fig. 4b). At this time *Cox10*/DAT-cre mice treated with pioglitazone performed better in the pole test than controls, being able to descend the pole in 30 % less time than *Cox10*/DAT-cre mice fed with standard chow (Fig. 4c). After 4 months of treatment, their performance was still improved (36 % better than untreated), though it did not reach the levels of control DAT-cre mice, (Fig. 4c).

When tested in the open field for locomotion and spontaneous activity, *Cox10*/DAT-cre mice showed improvement already after 2 months of treatment in travelled distance (Fig. 4d), ambulatory counts (Fig. 4e) and, most importantly, vertical counts (Fig. 4f) as rearing activity is highly dependent on dopaminergic innervation [32]. Remarkably, at 4 months of pioglitazone treatment improvement was still maintained, (Fig. 4f). Because neurodegeneration at this time (6 months of age) is almost complete, with virtually no TH+ cells remaining in the *substantia nigra*, this improvement is phenotypically meaningful.

**Fig. 5:**
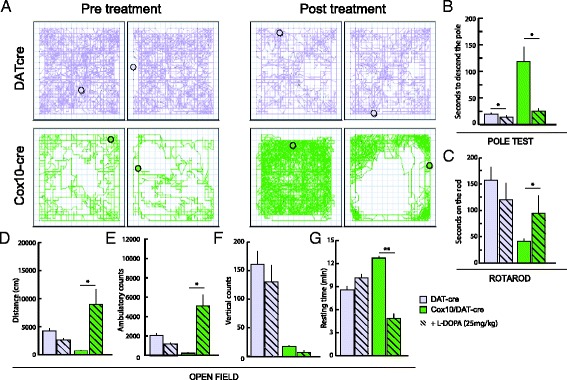
*Cox10* deficient mice display abnormal motor behaviors in response to L-DOPA treatment at 6 months of age. **a**, **d**-**g** Open field test: *Cox10*/DAT-cre mice show a reduced voluntary movement and decreased rearing activity compared to DAT-cre animals (*n* = 4), one hour after L-DOPA treatment (25 mg/kg) they show stereotyped movement **a**, hyperactivity **d**, **e**, and decreased resting time **g**. **b** Pole test: after L-DOPA treatment, *Cox10*/DAT-cre mice displayed decreased latencies times when required to descend a pole (*n* = 4). **c**: Rotarod test: after L-DOPA treatment *Cox10*/DAT-cre mice show increased coordination on staying on a rotating rod (*n* = 4)

To test whether at 6 months old *Cox10*/DAT-cre mice are still responsive to L-DOPA, like 2-month old mice were, we treated *Cox10*/DAT-cre and DAT-cre mice with three increasing doses of L-DOPA (6.25 mg/kg, 12.5 mg/kg and 25 mg/kg) in combination with benserazide. At low doses L-DOPA was not able to attenuate the motor deficits, but at the higher dose (25 mg/kg) it completely rescued the phenotype on the pole (Fig. 5b) and rotarod (Fig. 5c) tests. Nevertheless, as seen in younger mice, at 6 months of age, *Cox10*/DAT-cre mice showed atypical motor behavior (Additional file 1: Movie 1), with hyperactivity (Fig. 5a, d-g) and abnormal stereotypic movements (running along the borders of the cage) (Additional file 1: Movie 1, Fig. 5a). This atypical reaction has been described also in tyrosine hydroxylase KO mice, which are incapable of producing dopamine [33]. This behavior was absent in *Cox10*/DAT-cre mice (or controls) treated with pioglitazone (Fig. 4).

### Pioglitazone treatment decreases neuroinflammation in the *Cox10*/DAT-cre mice

**Fig. 6:**
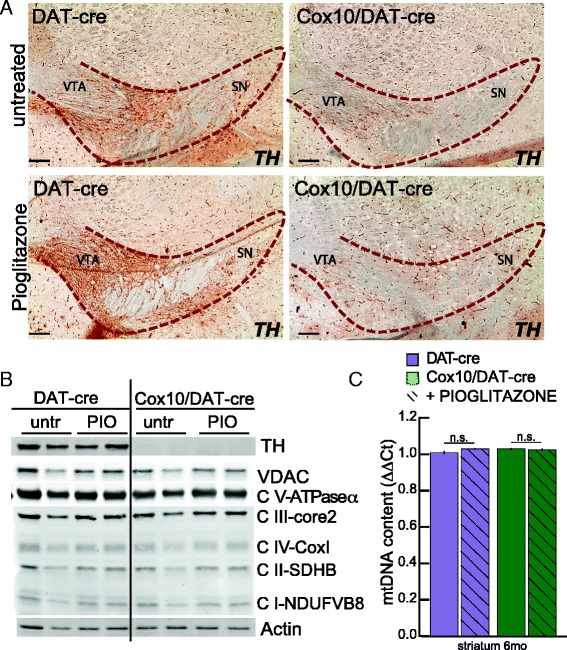
Pioglitazone treatment does not affect neurodegeneration or mitochondrial biogenesis. **a** immunohistochemistry with antibody anti-TH on DAT-cre and *Cox10*/DAT-cre mice untreated or treated with pioglitazone. Pioglitazone treatment does not affect the neurodegeneration. Red dashed line surrounds the *substantia nigra*. **b** western blot performed on striatal protein homogenates of DAT-cre and *Cox10*/DAT-cre mice untreated (untr) or fed with pioglitazone (PIO), with markers of dopaminergic axons (TH) and mitochondrial markers (VDAC-porin, NDUFB8 (CI subunit), SDHB (CII subunit), UQCRC2 (core2 subunit of CIII), COXI (CIV subunit) and ATPase-α (CV subunit). **c** mtDNA content measured by qPCR in striatal samples of DAT-cre and *Cox10*/DAT-cre mice untreated or treated with pioglitazone

Pioglitazone has been shown to prevent drug-induced neurodegeneration in mammalian animal models [22, 23]. In order to understand if the effect of pioglitazone on *Cox10*/DAT-cre mice was due to preventing or to slowing down dopaminergic neurodegeneration, we analyzed TH+ cells in the *substantia nigra* (Fig. 6a)*.* We did not detect changes in the essentially absent TH+ cells in the midbrain of *Cox10*/DAT-cre mice treated with pioglitazone.

We also analyzed striatal axonal degeneration by measuring TH protein expression, and found it to be unchanged in treated mice compared to controls in both genotypes (Fig. 6b). This is not unexpected, since neurodegeneration in these mice is already very severe at 2 months of age when treatment is initiated, and suggests that mechanisms other than preservation of dopaminergic neurons must be at the basis of pioglitazone therapeutic effect.

Because pioglitazone has been reported to increase mitochondrial biogenesis, we measured the steady-state levels of mitochondrial proteins in striatal homogenates, but did not detect any differences in the proteins tested (Fig. 6b). To further analyze mitochondrial biogenesis, we measured mtDNA content in the striatum and, similarly, did not detect any significant change between the groups (Fig. 6c).

**Fig. 7:**
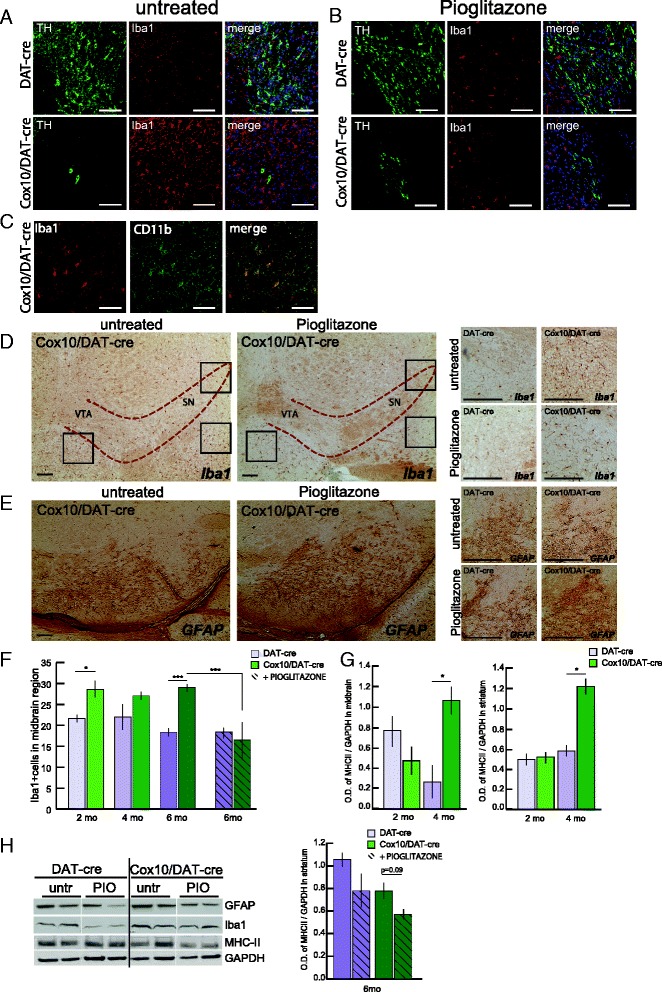
Pioglitazone treatment decreases the rate of neuroinflammation in the midbrain and striata of *Cox10*/DAT-cre mice. **a**-**b** double immunohistochemistry with antibody anti-TH and anti-Iba1 on midbrain slides of DAT-cre and *Cox10*/DAT-cre mice untreated **a** and treated with pioglitazone for 4 months **b**. Iba1+ cells are numerous in *Cox10*/DAT-cre midbrain sections and comparable to DAT-cre mice after pioglitazone treatment. Scale bar = 50 μm. **c** double immunohistochemistry with antibody anti-Iba1 and anti-CD11b (marker of activated microglia) on midbrain slides of 6 months old untreated *Cox10*/DAT-cre mice. Scale bar = 50 μm. **d** Immunohistochemistry with Ab anti-Iba1 on midbrain slides of *Cox10*/DAT-cre mice untreated and treated with pioglitazone. Red dashed line surrounds the *substantia nigra*. Black squares indicate the three regions chosen for Iba1+ cells counting. Enlargement of left black square shows numerous Iba1+ cells in *Cox10*/DAT-Cre mice. Scale bar = 50 μm. **e** Immunohistochemistry with Ab anti-GFAP on midbrain slides of *Cox10*/DAT-cre mice untreated and treated with pioglitazone. Enlargement shows no change of GFAP+ cells in *Cox10*/DAT-Cre mice. Scale bar = 50 μm. **f**: Microglial cells counting shows an increase of Iba1+ cells in *Cox10*/DAT-Cre midbrain at 2 and 6 months of age and a decrease after pioglitazone treatment (*n* = 3-6/group). **g**: Western blot and relative quantification of MHC-II, marker of activated microglia, in midbrain and striata of 2 and 4 months old DAT-cre and *Cox10*/DAT-cre mice (*n* = 3/group). **h**: Western blotting probing for markers of neuroinflammation (GFAP, Iba1, MHC-II) in striatal homogenates of DAT-cre and *Cox10*/DAT-cre mice untreated (untr) or treated with pioglitazone (PIO). Quantification of MHC-II normalized for GAPDH shows a decrease of glial activation after treatment with pioglitazone (*n* = 3/group)

A powerful effect of pioglitazone, demonstrated not only in the MPTP model of PD but other neurodegenerative diseases as well, is the ability to reduce neuroinflammation mainly by activating PPARγ in microglia [22, 34]. To test whether pioglitazone might be inhibiting the inflammatory response in our model, we analyzed microglial activation in both midbrain and striatum. In the midbrain of untreated *Cox10*/DAT-Cre animals we detected markedly increased immunoreactivity for Iba1 compared to control mice (Fig. 7a) but not an increase in GFAP immunoreactivity (Fig. 7e). We also confirmed by western blot that GFAP in midbrain of 6-month-old *Cox10*/DAT-Cre was not altered (Additional file 2: Figure S1A-B). To confirm that the cells were microglial cells, we also used anti-cd11b antibody, a second marker of microglia (Fig. 7c). As further confirmation of microglial activation, we detected a significant increase in the expression of MHCII in midbrain homogenates of 4 months old animals compared to controls (Fig. 7g).

When we analyzed *Cox10*/DAT-cre mice after 4 months of treatment with pioglitazone (6 months of age), we found a dramatic reduction in Iba1 immunoreactivity in the midbrain compared to untreated animals (Fig. 7b, d), and when we quantified the number of Iba + cells in pioglitazone-treated *Cox10*/DAT-cre animals we found them to be comparable to DAT-cre controls (Fig. 7f). In contrast, when we analyzed striatal samples, we did not find any increase in Iba1 + cells or in Iba1 content (data not shown), but we detected increased MCHII levels in 4 months old animals (Fig. 7g).

The content of Iba1 in the striatal homogenates of the mice treated with pioglitazone did not change significantly (Fig. 7h), but the content of MHCII showed a trend towards a decrease in pioglitazone-treated mice compared to untreated *Cox10*/DAT-cre animals (Fig. 7h).

To further investigate the inflammatory response, we measured the steady-state levels of inflammatory cytokines by multiplex technology in midbrain and striatum. IL1β, IL6, Il10, CXCL10 and CCL2 showed a trend to be higher in the striatum of *Cox10*/DAT-cre mice already at 2 months of age, but the differences were not significant (Additional file 2: Figure S1C). All, except IL-10 also showed a trend towards an increase in the midbrain, with IL1β, IP10 and MCP1 reaching significance (Additional file 2: Figure S1D). We did not detect an increase in older ages (Additional file 2: Figure S1E-F). In pioglitazone-treated animals, all the cytokines analyzed trended towards lower levels in *Cox10*/DAT-cre mice, but only MCP1 was significantly reduced (Additional file 2: Figure S1G). CCL5 and TNFα were also analyzed but were undetectable in all samples.

## Discussion and conclusions

The creation of animal models is crucial to study the pathogenesis of PD and to develop new therapies. Although there is no perfect model that encompasses all characteristics of PD pathogenesis, each allow specific aspects of the disease to be analyzed. Drug-induced models (6-OHDA, MPTP, Rotenone) cause fast degeneration of dopaminergic neurons and can be used, for example, to test neuroprotective drugs, usually administered before the toxin itself. MPTP crosses the blood–brain barrier and its metabolite, MPP+, blocks Complex I of the electron transport chain, inducing rapid neurodegeneration of the dopaminergic neurons. However, MPTP has several disadvantages: 1) its extreme toxicity makes it difficult and dangerous to work with; 2) the reproducibility of the lesion depends on the mouse strain, gender, age and body weight [35], as well as on the purity of the compound and on the administration protocol; 3) this treatment has a high death rate which occurs within the first 24 hours and is unrelated to the damage in the dopaminergic system [36]. 6-OHDA is also effective in inducing dopaminergic lesions, but has to be focally injected into small brain regions [37], causing variability in the results.

Although most cases of PD are sporadic, to study the pathophysiology of this disease mouse models have been created by knocking-out or knocking-in mutated forms of genes that are involved in the rare genetic forms of PD [38]. Among them, mice overexpressing α-synuclein or mutated forms of LRRK2 [39], and knockout for Parkin, PINK1 and DJ1 have been created. Unfortunately, they showed only mild motor coordination defects and impairment in striatal DA release with no dopaminergic neurodegeneration. Nonetheless, they have been useful models to increase our understanding of the endogenous role of these gene products [40].

Because of the putative mitochondrial involvement in PD, transgenic mouse models have been created by disrupting mitochondrial functions and dynamics, two of them by reducing mtDNA levels: the “Mito-Park” mouse [41] and the “PD-mito-*Pst*I” mouse [28]. These mice show different rates of neurodegeneration, depletion of DA in the striatum, L-DOPA sensitive-defects in motor coordination, and abnormal mitochondrial aggregates, further indicating the importance of mitochondrial function in the dopaminergic system. Retrograde neurodegeneration has been induced also by knocking out Mfn2 in dopaminergic cells [32].

However, some mitochondrial perturbations do not induce neurodegeneration. For example, even though *Ndufs4*-KO animals (Complex I deficient) showed a severe phenotype and died before 7 weeks of age, knocking out *Ndufs4* only in dopaminergic neurons led to a mild reduction of dopamine in the striatum and did not induce neurodegeneration or motor defects [42].

We created a novel mouse model of PD by disrupting cytochrome c oxidase (Complex IV) in dopaminergic neurons. Dopaminergic axons in the striatum were markedly decreased in *Cox10*/DAT-cre mice at 2 months of age resulting in almost complete depletion of striatal dopamine. Accordingly, the number of TH+ neurons was also dramatically decreased at this time point with *Cox10*/DAT-cre mice showing a severe motor phenotype with a loss of coordination and a decrease in voluntary movement.

Further decline in motor coordination was observed between 2 and 4 months of age, but despite these defects, the life span of the mice was not markedly reduced and KO mice could be identified only by the decreased body weight. Although it is surprising that mice can live relatively normal lives in cages with such a severe loss of dopaminergic neurons, motor phenotypes were clear. Still, it points to important differences between the mouse and human disorders. Also, compared to other mouse models of PD, such a dramatic dopaminergic neurodegeneration has been obtained only with drug-induced models.

Because in our model, a massive neurodegeneration is observed, we could test the efficacy of different compounds in a model of advanced disease. In fact, when PD patients are diagnosed, typically they already show a loss of approximately 70 % of their dopaminergic neurons, and therefore current treatments are administered only to attenuate and slow the progression of the motor symptoms. Even though L-DOPA administration remains the most effective treatment in alleviating the motor symptoms of PD, its long-term use leads to LID L-Dopa-Induced Dyskinesia (LID) [43].

Pioglitazone is a PPARγ agonist traditionally used as an insulin-sensitizing drug for the treatment of type-II diabetes. Therefore, one of the advantages of this drug is that it is already used in a clinical setting. PPARγ is ubiquitously expressed in the CNS [34], including in TH+ dopaminergic cells in the *substantia nigra* and in the *ventral tegmental area* [44].

This transcription factor regulates gene-expression programs of metabolic pathways and its activation is also involved in increasing expression of mitochondrial proteins, enhancing mitochondrial function and OXPHOS capacity [45]. PPARs also have a role in inflammation and in neurodegenerative disorders that have a inflammatory component, including PD, AD, stroke, ALS and spinal cord injury [46]. In recent studies, it has been shown that pre-treatment of non-human primates with pioglitazone protects dopaminergic neurons from MPTP-induced neurodegeneration, modulating inflammation and increasing the expression of PPAR-γ [20, 22]. However, it is important to note that a very recent phase II clinical trial of PD patients with pioglitazone, though inconclusive, did not suggest a major beneficial effect [47].

Here we report the beneficial effects of pioglitazone on the motor phenotype of a mouse model of PD with early-onset dopaminergic neuron loss due to a mitochondrial respiratory chain defect. In contrast to the other studies, pioglitazone was administered when the vast majority of TH+ neurons had already degenerated, a scenario akin to PD patients at diagnosis. The positive effect of pioglitazone on motor behavior was not sufficient to restore the motor coordination to control levels, but this is not surprising, considering that the neurodegeneration in the *Cox10*/DAT-cre mice was already severe at 2 months of age.

One possible mechanism for an amelioration of the motor symptoms could be a decrease in apoptosis of dopaminergic neurons by decreasing the activity of caspase-3 [48]. However, we did not detect any change in neuronal loss of treated mice compared to controls probably because of the already massive degeneration that affects *Cox10*/DAT-cre mice at this young age. A second hypothesis could be that, even if the neurodegeneration is not decreased, there is a stimulation of mitochondrial biogenesis by the action of pioglitazone in activating PGC-1α and other mitochondrial genes in the remaining cells. However, we did not detect changes in mitochondrial biogenesis. As mentioned before, one of the mechanisms involved in pioglitazone-mediated neuroprotection is the decrease of neuroinflammation. Indeed, our results are in agreement with this model. We analyzed inflammation in the two brain regions mostly affected by dopaminergic neurodegeneration and we detected differences in neuroinflammation: Iba1+ cells were increased in the midbrain but not in the striatum of *Cox10*/DAT-cre animals, while MHCII, a marker of activated microglia, was increased in both regions in 4-month-old animals. This suggests that consequential inflammation occurs in the midbrain but also, to a lesser extent, in the striatum by activation but not proliferation of the resident microglial cells. Moreover, the activation of microglia is concomitant with the worsening of the motor phenotype at 4 months. IL1β, CXCL10 and CCL2 were significantly increased in the midbrain of 2 month-old *Cox10*/DAT-cre mice compared to DAT-cre animals. Even if not significant, we also detected a trend of all other cytokines to be increased in striatum and midbrain of 2 and 6-month-old *Cox10*/DAT-cre animals compared to DAT-cre mice. Pioglitazone treatment specifically reduced both microglial cell number in the midbrain and microglial activation in midbrain and striatum. Moreover, CCL2 was significantly decreased in the striatum of *Cox10*/DAT-cre mice treated with pioglitazone.

It is possible that pioglitazone may benefit only cases where neuroinflammation is prominent, which could be restricted to a sub-population of PD patients, or that the effect of pioglitazone may be stage-dependent, as neuroinflammation becomes more prominent as the disease progresses [49]. This would explain the inconsistent results with patients [47].

Our mouse model also showed another interesting phenotype. When we attempted to compare the improvement in motor coordination obtained with pioglitazone treatment with age-matched mice (6 months of age) treated with L-DOPA, we noticed hyperactivity, abnormal stereotypic movements and absence of rearing activity. Dysregulation of DA release and clearance resulting from a loss of nigrostriatal DA terminals [50] is also involved in the development of LID. LID is one of the main side effects of L-DOPA treatment in PD patients, who develop LID in 90 % of cases after 8–10 years of L-DOPA treatment. The molecular mechanism responsible for the development of LID is still not fully characterized, and the most utilized animal model for this condition is the 6-OHDA-lesioned mouse, where the massive neurodegeneration is achieved by intracranial unilateral injection of 6-OHDA. Although further characterization would be necessary, the *Cox10*/DAT-cre mice also appear to show LID, and they can be potentially used as a model of late stage of PD with dyskinesia-resembling phenotype. Moreover, LID was not observed in mice treated with pioglitazone, suggesting a potential co-adjuvant, which may allow for reduced doses of L-DOPA for long-term PD patients.

## Methods

### Mice procedures

All animals used in this work were males and had a pure C57Bl/6 J background, backcrossed for at least 10 generations. All experiments and animal husbandry were performed according to a protocol approved by the University of Miami Institutional Animal Care and Use Committee. Mice were housed in a virus-antigen-free facility of the University of Miami Division of Veterinary Resources in a 12-h light/dark cycle at room temperature and fed ad libitum.

### Enzymatic activity assays

Striatum homogenates were prepared in PBS containing complete protease inhibitor cocktail (Roche diagnostics) in a volume of 10x the weight. The tissue was disrupted by 10–15 strokes, using a motor-driven pestle. Homogenates were centrifuged at 1000 g for 5 min and supernatants used for enzymatic assays. The activities of CIV and citrate synthase were measured spectrophotometrically as described [51]. Protein concentrations were determined using the Bio-Rad Bradford Assay Kit with bovine serum albumin (BSA) as standard. Specific activity was determined and values represented as μmoles/min/mg protein.

### Motor behavioral tests

#### Pole test

Pole test for motor coordination/nigrostriatal dysfunction of mice was previously described [52]. Animals were hung upright on a vertical (8 mm diameter; 55 cm length) pole and were given three minutes to change orientation to descend. Animals were given three trials with an average taken of the latency to descent to the base. Failure to descend or fall from the pole was given a maximum time of three minutes.

#### RotaRod test

Motor coordination was evaluated with a RotaRod (IITC Life Sciences) designed for mice. Animals were tested with five runs on a given day with one run for practice. Three runs were recorded and combined to find the average latency to fall. A resting period of 120 seconds between each run was given. Animals were required to position limbs to stay on a rotating rod accelerating from 6 rpm-20 rpm over a 180 seconds time period. Mice that completed the task received a final latency time of 180 seconds.

### Ambulatory movement measurement

Spontaneous self-initiated movement was recorded using an activity cage setup (Columbus Instruments) designed for mice. Animals were housed individually in a novel cage environment thirty minutes prior to their dark cycle and monitored for a twelve or twenty-four hour period undisturbed. Ambulatory movement was counted as the number of infrared beam breaks that occurred inside the cage.

### Open field test

Open field (Med Associates Inc.) is a sensitive method for measuring gross and fine locomotor activity. It consists of a chamber and a system of 16 infrared transmitters that record the position of the animal in the three dimensional space. With this system not only the horizontal movement can be recorded but also the rearing activity. For our study, the animals were placed in the chamber 30 minutes before the test and the locomotor activities were recorded for 15 minutes.

### Pharmacological treatment

Mice were treated with L-3,4-dihydroxyphenylalanine (L-DOPA) at different concentrations (see manuscript) and benserazide (3,125 mg/kg, Sigma) dissolved in saline, administrated via intraperitoneal injection (I.P.) one hour before behavioral testing.

Pioglitazone food was provided by Bio-Serv (Frenchtown, NJ). Pharmaceutical Actos tablets (pioglitazone) were incorporated into the mouse diet at a concentration of 120 mg/kg.

### Dopamine and metabolite quantification

The Vanderbilt University CMN/KC Neurochemistry Core Lab using HPLC separation followed by fluorescent and/or electrochemical detection performed dopamine and metabolite quantification measurements. Freshly isolated striatum was harvested and quickly frozen in liquid nitrogen from mice sacrificed as previously described.

### Western blots

Protein extracts were prepared from the striatal neuroanatomical regions and homogenized in PBS containing a protease inhibitor mixture (Roche). Upon use, SDS was added to the homogenate at the final concentration of 4 %. Homogenates were then centrifuged at 14,000 g at 4 °C, and the supernatant was collected for analysis. Protein concentration was determined by Lowry assay using the BCA kit (BioRad). Approximately 30–50 μg of protein was run on a 4-20 % gradient Tris–HCl gel (BioRad) and transferred to a PVDF or nitrocellulose membrane (BioRad).

Membranes were blocked in 1:1 Odyssey blocking solution (LI-COR Biosciences) for 1 h at room temperature. Primary antibodies, which were incubated overnight at 4 °C, were: anti-TH (tyrosine hydroxylase) 1:1000 dilution (Sigma), anti-DAT (Dopamine transporter) 1:1000 (Sigma), anti-porin/VDAC 1:2000 (MitoSciences), OXPHOS cocktail rodent mixture 1:1000 (MitoSciences), α-tubulin 1:2000 (Sigma), β-actin 1:5000 (Sigma), GFAP (glial fibrillary acidic protein) 1:1000 (Cell Signaling Technology), Iba1 1:500 (Wako), MHCII 1:1000 (Abcam), NeuN 1:1000 (Chemicon), TUJ1 1:1000 (abcam). Secondary antibodies used were infrared-conjugated antibodies anti-rabbit-700/anti-mouse-800 (Rockland) at 1:3000 to 1:5000 concentrations respectively, and incubated at room temperature for 1 h. Blots were visualized with Odyssey Infrared Imaging System (LI-COR Biosciences). Optical density measurements were taken by software supplied by LI-COR.

### Immunostaining

Anesthetized mice were transcardially perfused with ice-cold PBS and 4 % PFA. The brain was isolated, regions of interest were dissected using a brain matrix, and cryoprotected in sucrose 30 % and frozen in OCT. Frontal sections were cut at a 20-μm thickness with a cryostat (Leica).

Sections were blocked with 10 % normal goat serum (NGS) for 30 min at RT, and then incubated with primary Ab for 16 h at 4 °C: anti-TH 1:500 (Sigma); GFAP 1:500 (Cell Signaling Technology), Iba1 1:500 (Wako) NeuN 1:500 (Chemicon), TUJ1 1:500 (abcam),. Slides were then incubated with secondary Ab biotin-conjugated goat anti-mouse (KPL) for 1 h at RT and Streptavidin-Peroxidase (KPL) for 30 min at RT. Staining was visualized using a solution of 0.05 % 3,3’-diaminobenzidine (DAB), 50 mM Tris–HCl pH 7.2, 0.02 % H_2_O_2_. Images were captured with an Olympus BX51 microscope.

For immunofluorescent staining, sections were blocked with 10 % normal goat serum (NGS) for 1 h at RT, and permeabilized with 1 % Triton X-100. Sections were incubated with primary Ab (anti-TH 1:500 (Sigma), anti Iba1 1:500 (Wako)) for 16 h at 4 °C. Slides were then incubated with Alexa-fluor secondary Ab for 1 h at RT and mounted with Vectashield mounting medium for fluorescence. Images were captured with an Olympus BX51 confocal microscope.

### MtDNA content measurement by qPCR

DNA was isolated from the striatal homogenates using phenol:chloroform extraction. We designed a couple of primers to amplify mtDNA. To determine relative quantity of mtDNA in each sample, we used the comparative Ct method (Schmittgen and Livak, 2008), normalizing the amplification with primers that amplify a genomic DNA region. Relative quantity was corrected for relative PCR amplification efficiency using Biorad CFX Manager Software. Primers for mtDNA were as follows: ND1-3281 F, CAGCCTGACCCATAGCCATA; ND1-3364 B, ATTCTCCTTCTGTCAGGTCGAA. Primers for genomic DNA were as follows: β-actin F, GCGCAAGTACTCTGTGTGGA; β-actin B, CATCGTACTCCTGCTTGCTG. Targets were amplified using Maxima SYBR Green/ROX qPCR Master Mix (Fermentas) using CFX96 Realtime PCR system (Bio-Rad) under the suggested PCR conditions from the manufacturer.

### Cell counting

Stereological counting was used to quantify Iba1+ cells. Immunohistochemical identification of Iba1+ cells is described above. Microglial cells were counted with ImageJ program in double blind. Four sections were randomly chosen through the midbrain and three specific midbrain regions (depicted with black squares in Fig. 7) were chosen for the counting, with a total of 12 counting sections per individual animal. Iba1+ cell numbers were determined on 20 μm immunohistochemical stained sections. A guard zone of 3 μm from the top and from the bottom of each section was not considered in the counting. The midbrain area was identified using the 4× objective and Iba1+ cells were counted at 20x magnification.

### Multi-analyte profiling: determination of Cytokines in brain tissues

Concentrations of IL-1β, IL-6, IL-10, CXCL10 (IP10), CCL2 (MCP-1), CCL5 (RANTES), TNFα, were simultaneously quantified in each brain tissue sample using a MILLIPLEX®MAP mouse cytokine magnetic bead panel (Millipore). Briefly, brain tissues were collected and snap frozen in liquid nitrogen, homogenized in suggested lysis buffer (20 mMol TrisHCl pH7.4, 150 mMol NaCl, 1 mMol PMSF, 0.05 % Tween-20, protease inhibitor mixture), sonicated for 5” and centrifuged for 10’ at 10000 g. Supernatant was collected and protein concentration was adjusted to 1 mg/ml and diluted 1:1 with sample buffer. Plate was prepared following the manual instructions. Cytokine concentrations were then measured using MagPix Luminex100 reader and analyzed by Miami Center for AIDS Research (CFAR). Mean fluorescent intensities (MFI) were analyzed with MILLIPLEXTM Analyst Software (EMD Millipore) and cytokine levels expressed as pg/ml. CCL5 and TNFα were below detectable levels.

### Statistical analysis

Two-tailed, unpaired Student t-test was used to determine the statistical significance between two different groups. Multiple groups were compared using a one-way ANOVA followed by a Bonferroni post-hoc comparison. Error bars represent SEM. *P* values are indicated by asterisks (*p < 0.05, **p < 0.01, ***p < 0.001).

## Additional files

Additional file 1: Movie 1. High doses of L-DOPA rescued the phenotype of Cox10/DAT-cre mice, but also triggered atypical motor behavior, with hyperactivity and abnormal stereotypic movements. (MP4 33544 kb) Additional file 2: Figure S1. Pro-inflammatory cytokines are increased in Cox10/DAT-cre mice and decrease after Pioglitazone treatment. (PDF 277 kb)

## Abbreviations

3-MT: 3-methoxytyramine
6-OHDA: 6-hydroxydopamine
AD: Alzheimer’s disease
ALS: amyotrophic lateral sclerosis
CCL2 (MCP-1): C-C motif chemokine ligand 2 (monocyte chemotactic protein 1)
CCL5 (RANTES): C-C motif chemokine ligand 5 (regulated on activation, normal T cell expressed and secreted).
CIV: complex IV
CS: Citrate synthase
CXCL10 (IP-10): C-X-C motif chemokine ligand10 (Interferon gamma-induced protein 10)
DA: dopamine
DAT: dopamine transporter
DOPAC: dihydroxyphenylacetic acid
HVA: homovanillic acid
IL-1b: IL-6, IL-10, Interleukin 1b, 6, 10
L-DOPA: L-3,4-dihydroxyphenylalanine
LID: L-Dopa-Induced Dyskinesia
MHCII: Major histocompatibility complex, classII
MPTP: 1-methyl-4-phenyl-1,2,3,6-tetrahydropyridine
PD: Parkinson’s disease
PGC-1α: peroxisome proliferator-activated receptor gamma coactivator 1-alpha
PPAR-γ: peroxisome proliferator-activated receptor γ
SN: substantia nigra
TH: tyrosine hydroxylase
TNF-α: tumor necrosis factor-alpha
