# Supplementary material for: Pioglitazone ameliorates the phenotype of a novel Parkinson’s disease mouse model by reducing neuroinflammation
Source: Mol Neurodegener. 2016 Apr 2;11:25. doi: 10.1186/s13024-016-0090-7 (PMC4818913; doi:10.1186/s13024-016-0090-7)
Supplement: Additional file 2: Figure S1. — Pro-inflammatory cytokines are increased in Cox10/DAT-cre mice and decrease after Pioglitazone treatment. (PDF 277 kb) [file 13024_2016_90_MOESM2_ESM.pdf]

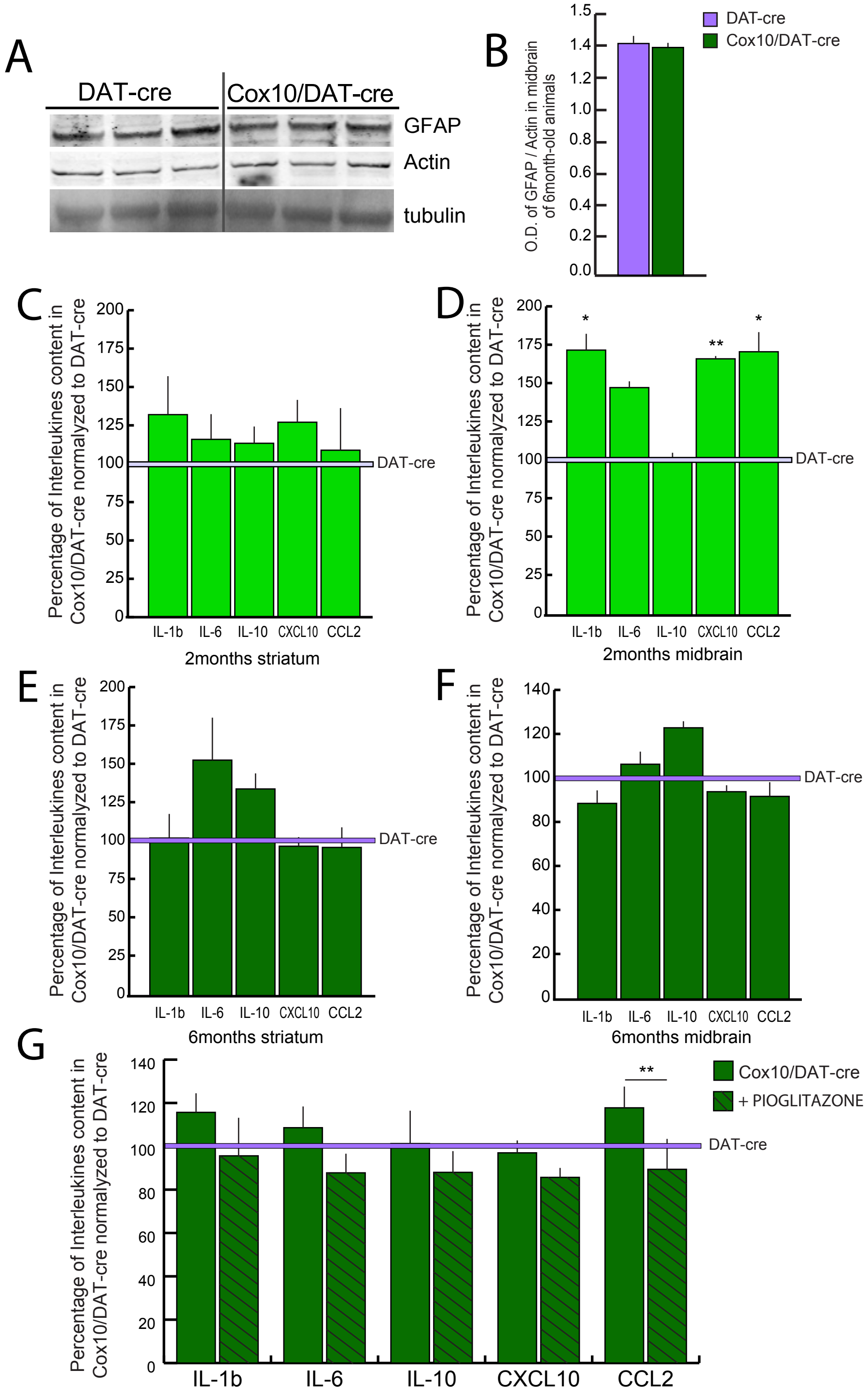


**Supplementary figure S1. Pro-inflammatory cytokines are increased in Cox10/DAT-cre mice and decrease after Pioglitazone treatment.**

A: Western blotting probing for markers of neuroinflammation GFAP in midbrain homogenates of DAT-cre and Cox10/DAT-cre mice. B: Western quantification shows no change in GFAP signal.

C-D: Percentage of pro-inflammatory cytokines content in striata (C) and midbrain (D) of 2-month-old Cox10/DAT-cre animals, normalized for cytokine content measured in DAT-cre mice.

E-F: Percentage of pro-inflammatory cytokines content in striata (E) and midbrain (F) of 6-month-old Cox10/DAT-cre animals, normalized for cytokine content measured in DAT-cre mice.

G: Percentage of pro-inflammatory cytokines content in striata of 6-month-old Cox10/DAT-cre animals untreated and treated with Pioglitazone, normalized for cytokine content measured in DAT-cre mice.
